# Expanding the Molecular Data of *DICER1*‐Related Tumor Predisposition: Novel Germline Variants in an Argentine Pediatric Cohort

**DOI:** 10.1155/humu/3621830

**Published:** 2026-09-30

**Authors:** Sofía Trobo, Natalia Pérez Garrido, Pablo Ramírez, Fiorella Tesan, Elisa Vaiani, Noelia Dujovne, Natalia Gazek, Jessica Lopez Martí, Mariana Lavia, Guido Felizzia, Victoria Pringles, Melisa Agotegaray, Nora Saraco, Marta Ciaccio, Alicia Belgorosky, Roxana Marino

**Affiliations:** ^1^ Endocrinology Molecular Laboratory Hospital de Pediatría Prof. Dr. Juan P. Garrahan Buenos Aires Argentina; ^2^ Endocrinology Service Hospital de Pediatría Prof. Dr. Juan P. Garrahan Buenos Aires Argentina; ^3^ Pathology Service Hospital de Pediatría Prof. Dr. Juan P. Garrahan Buenos Aires Argentina; ^4^ Genetics Service Hospital de Pediatría Prof. Dr. Juan P. Garrahan Buenos Aires Argentina; ^5^ Oncology Service Hospital de Pediatría Prof. Dr. Juan P. Garrahan Buenos Aires Argentina; ^6^ Consejo Nacional de Investigaciones Científicas y Técnicas (CONICET) Buenos Aires Argentina conicet.gov.ar

## Abstract

**Background/Objective:**

*DICER1*‐related tumor predisposition is a rare hereditary tumor predisposition disorder characterized by autosomal dominant inheritance, reduced penetrance, and variable expressivity. This study is aimed at describing the clinical and molecular spectrum of *DICER1*‐related tumor predisposition in an Argentine pediatric cohort.

**Design and Methods:**

Seventeen unrelated patients with *DICER1*‐related tumor predisposition and 54 relatives were evaluated at “Prof. Dr. J. P. Garrahan” Pediatric Hospital (Buenos Aires, Argentina) between 2014 and 2024. Germline and somatic *DICER1* variants were analyzed using Sanger or next‐generation sequencing. Functional assays were performed for selected intronic variants. Three samples of poorly differentiated thyroid carcinoma were also included.

**Results:**

Sixteen distinct germline variants were identified in the 17 index cases, including 12 novel alterations (75%). One quarter of the variants were de novo. Thyroid involvement, particularly multinodular goiter and papillary thyroid carcinoma, was the most common presentation and frequently coexisted with ovarian Sertoli–Leydig cell tumors or embryonal rhabdomyosarcoma. Two novel intronic variants were confirmed to affect splicing by RT‐PCR. Somatic “hotspot” mutations in exons 24–25 (RNase IIIb domain) were detected in most tumors, consistent with the two‐hit model. The identification of asymptomatic carriers enabled early surveillance and preventive interventions.

**Conclusions:**

This study expands the molecular spectrum of germline *DICER1* variants, reinforces the predominance of thyroid involvement, and highlights the value of molecular testing for diagnosis, genetic counseling, and early intervention. Ethical considerations regarding surveillance and management of asymptomatic carriers remain an important area in need of future guidance.

## 1. Introduction

*DICER1*‐related tumor predisposition is a rare familial tumor predisposition disorder caused by pathogenic germline variants in the *DICER1* gene, with an autosomal dominant inheritance pattern, reduced penetrance, and variable expressivity. Benign and malignant clinical manifestations occur predominantly in children and young adults. Beyond pleuropulmonary blastoma (PPB), the most common lung cancer in pediatrics [1, 2], the disorder is associated with a broad spectrum of neoplasms, including Sertoli‐Leydig cell ovarian tumor (SLCT), cystic nephroma (CN), female genitourinary embryonal rhabdomyosarcoma (FGU‐RMS), multinodular thyroid goiter (MNG), and thyroid carcinoma (TC). Additional, less frequent entities include anaplastic sarcoma of the kidney (ASK), nasal chondromesenchymal hamartoma (NCMH), pituitary blastoma (PitB), pineoblastoma (PinB), ciliary body medulloepithelioma (CBME), Wilms tumor (WT), intestinal juvenile hamartomatous polyps, and uncommon sarcomas of the cervix, kidney, and brain. Macrocephaly is observed in a significant proportion of patients and represents one of the few nontumor expressions of the disorder, together with certain structural kidney and ocular abnormalities [3–6].

The human *DICER1* gene locus is located on chromosome 14q32.13. Its product, the DICER1 protein, belongs to the type III ribonuclease family (RNase III) and is responsible for cleaving precursors of small noncoding RNAs (microRNAs or miRNAs) to generate mature miRNAs, which play a key role in regulating gene expression at the posttranscriptional level [7].

Individuals affected by *DICER1*‐related tumor predisposition carry a pathogenic germline variant in the *DICER1* gene. Although most correspond to inherited loss‐of‐function (LOF) changes, de novo variants have been described in approximately 10%–20% of cases [8, 9]. The vast majority of germline alterations are pathogenic LOF variants identified throughout the entire gene (Figure 1). However, other types of alterations have been documented, including deletions of the entire locus [8, 10–12], intragenic deletions involving one or more exons with and without frameshift [13, 14, 15], and somatic mosaicisms observed in a small proportion of syndromic patients [14, 16, 17].

**Figure 1:**
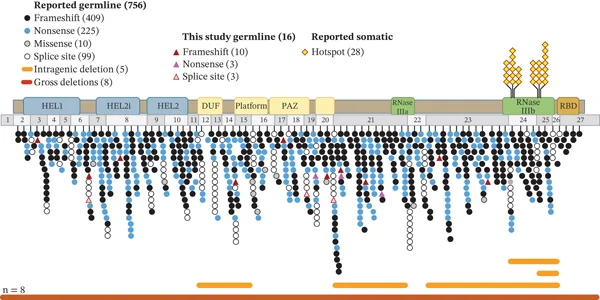
Plot of germline and somatic *DICER1* alterations represented along the length of the unfolded DICER1 protein. Pathogenic and likely pathogenic germline variants previously described in ClinGen database have been plotted below the protein. Sixteen germline variants reported in this study are also included. The 28 somatic pathogenic variants previously described most frequently associated with *DICER1*‐related tumor predisposition have been plotted above the protein. The vast majority of germline alterations are pathogenic LOF variants described throughout the entire gene sequence, whereas somatic variants are missense that involve any of the codons that encode metal‐binding residues or adjacent codons. (p.E1705, p.D1709, p.D1713, p.G1809, p.D1810, p.E1813) located in RNase IIIb domain (exons 24 and 25) a region considered as somatic hotspot.

*DICER1*‐related tumor predisposition associated tumors carry, in addition to the germline heterozygous pathogenic variant, a second somatic variant acquired in trans within the region encoding the RNase IIIb domain. This second somatic variant or second somatic “hit” consists of a missense variant that affects any of the codons that encode metal‐binding residues or adjacent codons (p.E1705, p.D1709, p.D1713, p.G1809, p.D1810, p.E1813) located in exons 24 and 25, a region considered as a somatic “hotspot” (Figure 1). In this particular way, *DICER1* fits the characteristics of a tumor suppressor gene, in which functional inactivation results from two mutational events leading to the loss of expression of a normal full‐length protein in the tumor: a pathogenic heterozygous germline variant and a second somatic mutation in the wild‐type allele [18].

In this study we report the clinical and molecular characterization of 17 families with *DICER1*‐related tumor predisposition followed at an Argentine tertiary hospital between 2014 and 2024. Molecular analysis of the *DICER1* gene identified 16 different germline variants, including 12 novel alterations. De novo variants were detected in 25% of index cases. The detailed characterization of the clinical phenotypes in both the index cases and relatives provides new insight into the spectrum of *DICER1*‐related tumor predisposition in a previously unreported pediatric population. In addition, the detection of asymptomatic carriers allowed the implementation of preventive measures in individuals without clinical manifestations at the time of diagnosis.

## 2. Methods

### 2.1. Patients

We studied 17 unrelated patients with *DICER1*‐related tumor predisposition who were evaluated in the Departments of Oncology, Endocrinology, and Genetics of the tertiary Pediatric Hospital “Prof. Dr. J. P. Garrahan” in Buenos Aires, Argentina. All patients had clinical suspicion of *DICER1*‐related tumor predisposition, either because they were recently diagnosed with a phenotype associated with the disorder or because they had been diagnosed in the past. A detailed family history of cancer was obtained for each case.

Written informed consent for the genetic study was obtained from all of the patients or their parents or guardians after a full explanation of the purpose and nature of all the procedures.

The study was approved by the Independent Ethics Committee of the “Prof. Dr. J. P. Garrahan” Pediatric Hospital (reference number: 830) on August 27, 2014.

### 2.2. Analysis of *DICER1* Germline Pathogenic Variants

Genomic DNA was isolated from peripheral blood leukocytes (PBL) of 17 index cases and 54 family members using standard procedures on the chemagic Magnetic Separation Module I (Chemagen, Baesweiler, Germany), following the manufacturer′s instructions. Germline samples from index cases 18 and 20 were analyzed at an external institution.

The entire coding region (exons 2–27) and flanking intronic splice sites of the *DICER1* gene were amplified by polymerase chain reaction (PCR) and sequenced using automated analyzers [6]. PCR products were purified with the QIAquick PCR Purification Kit (Qiagen, Buenos Aires, Argentina) and sequenced using the BigDye Terminator Version 3.1 Cycle Sequencing Kit (Applied Biosystems, Buenos Aires, Argentina) on an ABI PRISM 3130 Genetic Analyzer (Applied Biosystems, Buenos Aires, Argentina). The primers used for sequencing were the same as those used for PCR amplification.

Resulting nucleotide sequences were aligned to the GenBank reference sequence NC_000014.9 (GRCh38) using SeqScape software V0.2.6 (Applied Biosystems). Sequence variants were described according to the recommendations of the Human Genome Variation Society (http://www.hgvs.org/rec.html) using NCBI reference sequence NM_177438.3 for the *DICER1* transcript and NP_803187.1 for the corresponding DICER1 protein.

Resulting nucleotide changes were reconfirmed in each sample DNA by sequencing the antisense strand and by resequencing a newly generated PCR product from the original DNA extract.

Pathogenicity of germline variants was assessed through review of the published literature and interrogation of publicly available databases, including the National Center for Biotechnology Information (NCBI), the Database of Single Nucleotide Polymorphism (dbSNP), ClinVar, Ensembl Genome Browser, the Genome Aggregation Database (gnomAD), Varsome, the Human Gene Mutation Database (HGMD), Online Mendelian Inheritance in Man (OMIM), and the DICER1 Syndrome Information Network. A comprehensive evaluation of each variant was performed following the ClinGen DICER1 and miRNA‐Processing Gene Expert Panel Specifications to the ACMG/AMP Variant Interpretation Guidelines for DICER1, Version 1.4.0. All 16 germline variants found in this study have been submitted to ClinVar database (SCV: 007334822 to 007334837).

### 2.3. RT‐PCR

To determine the effect of the putative intronic variants (Families 3 and 10), total RNA from peripheral blood mononuclear cells was extracted from the index case and the relatives carrying the variant using TRIzol reagent (Sigma Life Science), according to the manufacturer′s instructions. Total RNA was reverse‐transcribed into cDNA using the SuperScript III Reverse Transcriptase system (Invitrogen, Buenos Aires, Argentina). RT‐PCR was performed to amplify *DICER1* transcripts using specific primers designed in our laboratory for each case (Family 3: forward ACCAAGAGTTTACTAAGCACC, reverse GTAAATGGTCCACAATCCACC; Family 10: forward ACACTGATCTTACCCCACTCA, reverse TCAACGTGGAGCTTACCAGG). The resulting PCR products were analyzed by gel electrophoresis and subsequently sequenced.

### 2.4. Analysis of *DICER1* Somatic Pathogenic Variants

Tumor samples were provided by the Pediatric Tumor Bank of the “Prof. Dr. J. P. Garrahan” Pediatric Hospital. DNA was extracted from fresh‐frozen tissue or cryobar freezing wells using the QIAamp DNA Mini Kit (QIAGEN) or formalin‐fixed paraffin‐embedded (FFPE) blocks using the QIAamp DNA FFPE Tissue Kit (QIAGEN) according to sample availability. All tissue samples were microscopically reviewed by a pathologist to confirm presence of tumor tissue.

Exons 24 and 25, which encode the catalytic RNase IIIb domain of the DICER1 protein and harbor the majority of the somatic pathogenic variants, were amplified by PCR and analyzed using Sanger sequencing.

### 2.5. NGS Sequencing

PBL genomic DNA from Patient 6 and tumor DNA from Patients 10, 14 and 15 were analyzed using a custom next‐generation sequencing (NGS) thyroid cancer panel.

Coding regions and intronic flanking sequences of the selected panel of genes were assessed by massively parallel sequencing with capture enrichment using a customized SureSelectXT Low Input strategy (Agilent Technologies, United States), following the manufacturer′s protocols. The resulting libraries were sequenced with 150 bp paired‐end reads on a MiSeq sequencer using the MiSeq Reagent Kit V2 (300 cycles) (Illumina, United States). More than 99% of the target regions were covered with a depth greater than 20x. Bioinformatic processing for single‐nucleotide variant detection was performed using a pipeline developed following the Broad Institute Genomic Analysis Toolkit (GATK) standards. The detection of copy number variants (CNVs) was performed using the DECON and panelcn.MOPS tools.

## 3. Results

### 3.1. Clinical Presentation

Table 1 lists the different families, their *DICER1*‐related tumor predisposition‐associated clinical phenotypes, and ages at presentation (P1–P17). We also included three tumor cases (P18, P19, P20) with a histopathological diagnosis of poorly differentiated TC (PDTC), an entity that in infancy is associated with nonsyndromic somatic *DICER1* variants acting as genetic drivers of the disease [19].

**Table 1:** Clinical phenotypes in index cases.

Family	Phenotype in index case	Age at diagnosis (year)	Sex	DICER1 spectrum tumors in other proband′s relatives
P1	MNG	11	F	No
P2	CN	2	M	Mother^a^ and cousin^a^: MNG; aunt^a^: OS; cousin^a^: MNG + SLCT
P3	MNG	12	M	No clear evidence
P4	SLCT; MNG	14; 14	F	Aunt and half‐sister^a^: MNG; niece^a^: MNG + SLCT
P5	SLCT; MNG	10; 10	F	Mother^a^: PTC; brother^a^: CN + MNG
P6	ASK	5	M	Aunt: uERMS; aunt: uERMS
P7	MNG	7	F	Father, uncle and grandfather: MNG
P8	PTC; LC	15	M	No
P9	MNG	7	F	Mother^a^, uncle and three cousins: MNG
P10	FGU‐RMS; PTC	13; 15	F	Mother^a^: WT + MNG; brother^a^: MNG
P11	MNG	15	M	No
P12	Bilateral SLCT, MNG	3 and 6;	F	No clear evidence
P13	MNG	12	F	Father^a^ and brother: MNG
P14	PTC	5	F	No
P15	PTC; SLCT	16; 16	F	No
P16	PinB	6	M	No clear evidence
P17	FGU‐RMS; MNG	14; 17	F	No
P18	*PDTC*	12 year and 10 months	F	No
P19	*PDTC*	12	M	Not available
P20	*NB*; *PDTC*	4; 9	M	No

*Note:* Tumors not associated with *DICER1*‐related tumor predisposition are indicated in italics.

Abbreviations: ASK, anaplastic sarcoma of the kidney; CN, cystic nephroma; FGU‐RMS, female genitourinary embryonal rhabdomyosarcoma; LC, lung cysts; MNG, multinodular goiter; NB, neuroblastoma; OS, ovarian sarcoma; PDTC, poorly differentiated thyroid carcinoma; PinB, pineoblastoma; PTC, papillary thyroid carcinoma; SLCT, Sertoli‐Leydig cell tumor; WT, Wilms tumor.

^a^Denotes relatives who have undergone genetic testing and tested positive for the familial germline variant.

Regarding the organs involved in the index cases, our cohort of patients showed a high frequency of thyroid manifestations (MNG and TC phenotypes) and female genital tract tumors (embryonic rhabdomyosarcomas and SLCT). In fact, all female index cases presented with clinical manifestations in the thyroid, the female genital tract, or both. Among 11 female probands, six developed combined phenotypes in the thyroid and genital tract (P4, P5, P10, P12, P15, P17) whereas the remaining five had thyroid involvement as their only manifestation (P1, P7, P9, P13, P14). Among the six male probands, three presented with thyroid disease (P3, P8, P11) whereas the remaining three developed other tumors, including CN (P2), ASK (P6), and PinB (P16). Interestingly, index case P11, diagnosed with MNG, underwent surgery to remove not only thyroid tissue but also a suspicious‐looking lymph node adjacent to the thyroid gland. Histological evaluation of this node revealed lymph node sarcoidosis (LNS).

Among the 17 index cases, six presented with MNG as the sole manifestation of the disorder (P1, P3, P7, P9, P11, P13), whereas papillary TC (PTC) appeared as the only manifestation in two cases (P8, P14).

Figure 2 illustrates the family histories associated with *DICER1*‐related tumor predisposition. Eight index cases had a documented family history of at least one clinical manifestation of the condition in one or more relatives.

**Figure 2:**
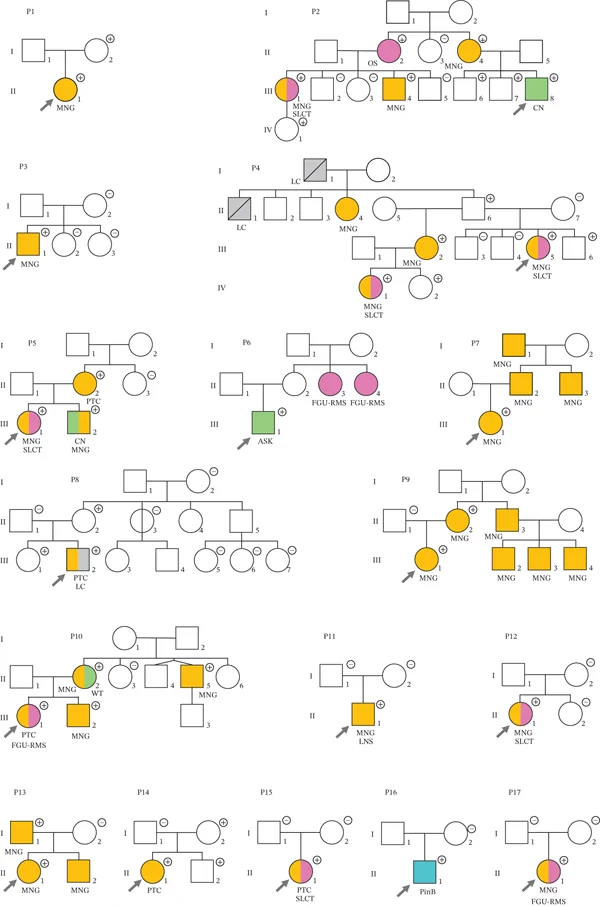
Pedigree of the 17 studied families. The index case is indicated by an arrow. ‘+’ and ‘−’denote mutation status (+ = mutation). Clinical phenotypes are color‐coded as follows: yellow for thyroid pathologies (MNG, PTC, LNS); pink for phenotypes of the female reproductive tract (FGU‐RMS, SLCT, OS); green for kidney pathologies (CN, ASK, WT); gray for lung diseases (LC); light blue for pineoblastoma (PinB); and white for asymptomatic individuals. ASK: anaplastic sarcoma of the kidney; CN: cystic nephroma; FGU‐RMS: female genitourinary embryonal rhabdomyosarcoma; MNG: multinodular goiter; LC: lung cysts; OS: ovarian sarcoma; PinB: pineoblastoma; PTC: papillary thyroid carcinoma; SLCT: Sertoli‐Leydig cell tumor; WT: Wilms tumor.

### 3.2. Molecular Characterization

As shown in Table 2, a heterozygous germline *DICER1* variant was identified in all 17 index cases. Family studies were performed in 54 relatives from 15 families. De novo germline variants were found in three of 12 (25%) index cases (P11, P15, P17) after ruling out the presence of the variant in both parents. In nine of 12 (75%), we were able to establish hereditary transmission by identifying the variant in at least one of the parents. Unfortunately, in the remaining five families, parental samples could not yet be obtained.

**Table 2:** Molecular data of germline and somatic variants in *DICER1* in index cases.

	Germline *DICER1* variant						Somatic *DICER1* variant				
Index case	cDNA	gDNA	Protein	Location	ACMG classification	Novel/reported	Tumor	cDNA	gDNA	Protein	Location
P1	c.202_205delinsAA	g.95132617_95132620delinsTT	p.(Ser68Lysfs ^∗^11)	Exon 3	LP (PVS1_vs, PM2_supp)	Novel	MNG	NA	NA	NA	
P2	c.731_734del	g.95129474_95129477del	p.(Asp244Glyfs ^∗^27)	Exon 6	P (PVS1_vs, PM2_supp, PP1_supp)	Novel	CN	NA	NA	NA	
P3	c.734+3A>G	g.95129469T>C	—	Intron 6	VUS (PS3_strong, PM2_supp)	Novel	MNG	c.5126A>G	g.95094126T>C	p.(Asp1709Gly)	Exon 24
P4	c.1052del	g.95124522del	p.(Phe351Serfs ^∗^2)	Exon 8	P (PVS1_vs, PM2_supp, PP1_supp, PP4_supp)	Novel	SLCT; MNG	c.5113G>A;	g.95094139C>T;	p.(Glu1705Lys);	Exon 24;
								c.5126A>G	g.95094126T>C	p.(Asp1709Gly)	Exon 24
P5	c.2222del	g.95111352del	p.(Gly741Valfs ^∗^17)	Exon 14	LP (PVS1_vs; PM2_supp)	Novel	SLCT; MNG	c.5437G>C;	g.95091293C>G;	p.(Glu1813Gln);	Exon 25;
								c.5113G>A	g.95094139C>T	p.(Glu1705Lys)	Exon 24
P6	c.2698dup	g.95107714dup	p.(Ile900Asnfs ^∗^2)	Exon 17	LP (PVS1_vs, PM2_supp)	Novel	ASK	NA	NA	NA	
P7	c.2987+1G>A	g.95106040C>T	—	Intron 18	VUS (PVS1_mod, PM2_supp, PS4_supp)	[20]	MNG	NA	NA	NA	
P8	c.3041T>G	g.95105730A>C	p.(Leu1014 ^∗^)	Exon 19	LP (PVS1_vs, PM2_supp)	Novel	PTC;	ND;	ND;	ND;	
							LC	NA	NA	NA	
P9	c.3242del	g.95105100del	p.(Gly1081Glufs ^∗^14)	Exon 20	LP (PVS1_vs, PM2_supp)	Novel	MNG	NA	NA	NA	
P10	c.3269+1G>A	g.95105070C>T	—	Intron 20	P (PVS1_vs, PM2_supp, PP4_supp, PP1_supp)	Novel	FGU‐RMS; PTC	c.5428G>T;	g.95091302C>A;	p.(Asp1810Tyr);	Exon 25
								c.5438A>T	g.95091292T>A	p.(Glu1813Val)	
P11	c.3282dup	g.95104114dup	p.(Asp1095Argfs ^∗^9)	Exon 21	P (PVS1_vs, PS2_supp,PM2_supp)	Novel	MNG; *LNS*	NA;	NA;	NA;	Exon 24
								c.5126A>G	g.95094126T>C	p.(Asp1709Gly)	
P12	c.3293G>A	g.95104103C>T	p.(Trp1098 ^∗^)	Exon 21	P (PVS1_vs, PM2_supp, PS4_mod)	[21]	bSLCT^a^; MNG	c.5113G>A;	g.95094139C>T;	p.(Glu1705Lys);	Exon 24
								c.5113G>A	g.95094139C>T	p.(Glu1705Lys)	
P13	c.3535_3538del	g.95103862_95103865del	p.(Ser1179Thrfs ^∗^12)	Exon 21	P (PVS1_vs, PM2_supp, PS4_mod)	[22, 23]	MNG	NA	NA	NA	
P14	c.3700G>T	g.95103696C>A	p.(Glu1234 ^∗^)	Exon 21	P (PVS1_vs, PM2_supp, PP4_supp)	Novel	PTC	c.5428G>T	g.95091302C>A	p.(Asp1810Tyr)	Exon 25
P15	c.4309_4312del	g.95096610_95096613del	p.(Asp1437Metfs ^∗^16)	Exon 23	P (PVS1_vs, PM2_supp, PS2_supp, PS4_mod)	[23–25]	PTC; SLCT	c.5113G>A;	g.95094139C>T	p.(Glu1705Lys);	Exon 24
								NA	NA	NA	
P16	c.4309_4312del	g.95096610_95096613del	p.(Asp1437Metfs ^∗^16)	Exon 23	P (PVS1_vs, PM2_supp, PS4_mod)	[23–25]	PinB	ND	ND	ND	
P17	c.4786del	g.95096137del	p.(Arg1596Glyfs ^∗^24)	Exon 23	P (PVS1_vs, PS2_mod, PM2_supp)	Novel	FGU‐RMS; MNG	NA;	NA;	NA;	
								NA	NA	NA	
P18	ND	ND	ND	—	—	—	*PDTC*	c.5437G>A	g.95091293C>T	p.(Glu1813Lys)	Exon 25
P19	NA	NA	NA	—	—	—	*PDTC*	c.5439G>C	g.95091291C>G	p.(Glu1813Asp)	Exon 25
P20	ND	ND	ND	—	—	—	*PDTC; NB*	c.5439G>T;	g.95091291C>A;	p.(Glu1813Asp);	Exon 25
								NA	NA	NA	

*Note:* Tumors not associated with *DICER1*‐related tumor predisposition are indicated in italics.

Abbreviations: ASK, anaplastic sarcoma of the kidney; bSLCT, bilateral SLCT; CN, cystic nephroma; FGU‐RMS, female genitourinary embryonal rhabdomyosarcoma; LC, lung cysts; LP, likely pathogenic; MNG, multinodular goiter; NA, not analyzed; NB, neuroblastoma; ND, not detected; OS, ovarian sarcoma; P, pathogenic; PDTC, poorly differentiated thyroid carcinoma; PinB, pineoblastoma; PTC, papillary thyroid carcinoma; SLCT, Sertoli‐Leydig cell tumor; VUS, variant of uncertain significance; WT, Wilms tumor.

^a^The tissue analyzed corresponds to the left ovary.

Of the 16 different germline variants identified, 13 were LOF exonic variants located in different exons of the gene (10 frameshift and three nonsense), whereas the remaining three were intronic variants. Twelve of the 16 variants (P1, P2, P3, P4, P5, P6, P8, P9, P10, P11, P14, P17) were novel (75%). Among these novel variants, two were nonsense (P8, P14), two were intronic (P3, P10), and eight were out‐of‐frame deletions or insertions that introduced a premature stop codon leading to truncation of the protein.

To analyze the two novel intronic variants (P3, P10), functional studies were performed using peripheral blood RNA from the index cases and their families. In P10, we detected a previously undescribed heterozygous intronic germline variant, NM_177438.3:c.3269+1G>A, which was also present in her mother and her brother, both of whom had the clinical phenotypes associated with the disorder. This variant was classified as pathogenic according to ACMG Guidelines (PVS1_vs, PM2_supp, PP4_supp, PP1_supp). As the variant was found in a splicing donor site in intron 20, cDNA from the patient, her mother, and a normal control was amplified using specific primers designed to amplify a 649 bp fragment between exons 18 and 21. As shown in Figure 3, an additional smaller fragment was detected in the agarose gel in both the patient and the mother, corresponding to a 473 bp product. Sequencing of this fragment confirmed complete skipping of exon 20, thereby demonstrating anomalous splicing.

In the case of P3, we found a previously undescribed heterozygous intronic germline variant, NM_177438.3:c.734+3A>G. Similar to P10, cDNA of P3 and a normal control were amplified. In this case, as the variant was located in intron 6, the primers were designed to amplify a 367 bp fragment between exons 4 and 7. We observed that in the index case, in addition to the fragment of the expected size of 367 bp, a smaller fragment of around 200 bp appeared, which when sequenced showed anomalous splicing with complete skipping of exon 6. Despite the direct impact on the splicing mechanism, causing a frameshift, according to ACMG Guidelines, this variant is classified as a variant of uncertain significance (PS3_strong, PM2_supp) (Figure 3).

**Figure 3:**
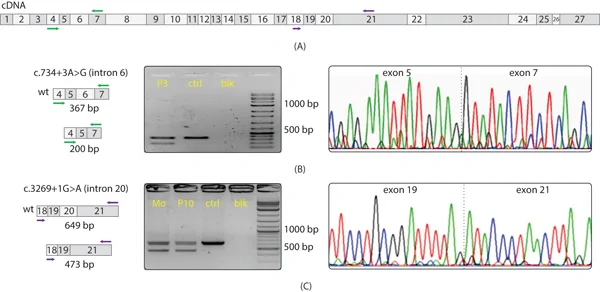
Functional assays for splicing disruption. (A), location of the primers in the *DICER1* cDNA sequence. (B), agarose gel electrophoresis: in the presence of the intronic variant c.734+3A>G, a smaller fragment is observed in the index case (P3) compared with the normal control. The electropherogram shows the complete skipping of exon 6. (C), in the index case (P10) and in her mother, the intronic variant c.3269+1G>A leads to abnormal splicing resulting in the complete loss of exon 20, as evidenced by sequencing the smaller fragment.

Tissue samples were available for somatic mutation analysis in 10 families. Molecular studies were performed in P3, P4, P5, P8, P10, P11, P12, P14, P15 and P16. In all cases except P8 and P16, we identified a previously described heterozygous somatic variant in the hotspot region of the gene. We detected eight somatic variants in exon 24 (in the MNG of P3, in the SLCT and MNG of P4, in the PTC of P5, in the LNS of P11, in the MNG and SLCT of P12, and in the thyroid tissue of P15) and five somatic variants in exon 25 (in the SLCT of P5, in the FGU‐RMS and PTC of P10, in the MNG of her brother, and in the PTC of P14).

Regarding the three tissues with histopathological diagnosis of PDTC, we identified a missense hotspot variant in exon 25 in all cases. To assess whether these variants were associated with *DICER1*‐related tumor predisposition, PBL DNA was analyzed in two out of three cases (P18, P20), as these were the only samples available. Sequence analysis ruled out the presence of a germline *DICER1* variant, confirming the nonsyndromic somatic nature of the variant (Table 2).

## 4. Discussion

Germline pathogenic variants in *DICER1* are known to predispose individuals to the development of multiorgan neoplastic and nonneoplastic conditions. Because the disorder is rare and was only relatively recently described, the number of patients and families reported to date is limited. Additional descriptions of cases are necessary to define and expand the clinical spectrum as well as the molecular data of the disease.

With respect to the affected tissues, we observed a marked predominance of thyroid gland involvement in our cohort. This agrees with the findings of Khan et al., who showed that three out of four women and one out of six men with *DICER1*‐related tumor predisposition would develop MNG by the age of 40 years [26]. A recent report also described that 13% of individuals under the age of 25 years diagnosed with nodular goiter based on thyroidectomy samples had pathogenic germline *DICER1* variants [27]. In our cohort, 14/17 index cases presented with a thyroid phenotype, of whom 12 were diagnosed with MNG (two of them later evolved to PTC follicular variant), and two were already diagnosed at the PTC stage (both follicular variants). These findings support previous evidence suggesting that nodular thyroid disease is likely the most common phenotypic manifestation in individuals with germline *DICER1* variants (particularly in females), with a higher penetrance than previously estimated [26–28].

An observation that drew our attention was that in five index cases (P1, P3, P7, P9, P13), MNG presented as the sole clinical manifestation of the disorder. In three of these families (P7, P9, P13), there was a clear history of relatives with a diagnosis of MNG as the only phenotype associated with *DICER1*. In the remaining two families, although the patients mentioned a family history of thyroid disease, we could not verify the accuracy of these data. We were not able to find an explanation for the high penetrance of MNG in these families, as the type and location of the germline variants were varied and, to our knowledge, no specific variant/phenotype correlation has been reported to date [18]. These observations are consistent with previous reports [26] suggesting that early‐onset MNG, MNG in a male patient, or MNG observed in several family members requires study of the *DICER1* gene, or at least a thorough investigation for disease/related manifestations in other family members. The frequent involvement of the thyroid gland in this pathology suggests a tissue‐specific susceptibility to DICER1 dysfunction. This phenomenon may be explained by the critical role of DICER1 in miRNA biogenesis, which is essential for maintaining thyroid follicular cell differentiation and homeostasis. Experimental models have demonstrated that DICER1 loss in thyroid tissue leads to disruption of follicular architecture and altered expression of key thyroid‐specific genes, supporting the notion that thyroid tissue is highly dependent on miRNA‐mediated regulation [18, 19]. Unlike many DICER1‐associated tumors that follow the classic two‐hit model in a focal manner, thyroid disease may reflect a unique process of multinodular evolution, whereby multiple independent RNase IIIb hotspot mutations arise in spatially distinct thyroid nodules over a germline *DICER1*‐mutated background, as demonstrated by deep sequencing studies of *DICER1*‐related MNG [16]. This model suggests that the thyroid gland may be particularly permissive to repeated clonal events, potentially contributing to the high penetrance of multinodular disease observed in *DICER1* carriers. At the same time, some thyroid lesions lack detectable somatic hotspot mutations, suggesting that partial *DICER1* dysfunction and haploinsufficiency may also contribute to thyroid hyperplasia in certain cases [8, 26]. Although these findings support plausible biological explanations for the predominance of thyroid manifestations, the mechanisms underlying the tissue‐specific susceptibility of the thyroid to DICER1 dysfunction remain incompletely understood and require further investigation.

It is also noteworthy that several individuals in our cohort presented with combined phenotypes involving both the thyroid and female genital tract. Among the female probands and their carrier relatives, eight fell into this category (P2, P4: index case and niece; P5, P10, P12, P15, P17). Six of them had MNG/PTC and SLCT phenotypes, whereas the remaining two showed combined MNG/PTC and FGU‐RMS (P10, P17). In this sense, our findings align with previous reports describing the same combination of phenotypes in carriers of germline *DICER1* variants [29–31]. The presence of these clinical phenotypes in the same individual, or even in different relatives within the same family, should raise clinical suspicion and lead to the consideration of germline *DICER1* testing.

Interestingly, patient P11, who was diagnosed with MNG, underwent surgery that included not only removal of thyroid tissue but also of a suspicious‐looking lymph node adjacent to the thyroid gland. Histopathological examination of the node revealed a diagnosis of LNS, an entity that, to our knowledge, has not been previously associated with this disorder. Molecular analysis of the lymph node′s cortical region revealed a hotspot variant in exon 24, raising the question of whether this represents a previously unidentified phenotype of the disease. However, more documented cases are needed to establish this link. It should be noted that our patient has not shown any signs of sarcoidosis affecting other organs during follow‐up.

As previously reported, approximately in 13% to 20% of individuals the detectable germline *DICER1* pathogenic variants occur de novo, whereas in 80% to 87% the variants are inherited [32]. In agreement with this, we identified de novo germline variants in 25% of the index cases whereas the remaining 75% showed hereditary transmission. We detected 16 different germline variants: 10 exonic frameshift variants and three nonsense variants, all of which result in a truncated protein with loss of its functional domains. The remaining three were intronic variants; two were novel and demonstrated aberrant splicing in functional assays. In both cases, the abnormal splicing resulted in a frameshift leading to a premature stop codon and, consequently, a nonfunctional protein.

The tumors associated with *DICER1*‐related tumor predisposition carry, in addition to the germline heterozygous pathogenic variant, a second somatic variant acquired in trans within the region encoding the RNase IIIb domain. This “second hit” consists of a missense variant that involves any of the codons that encode metal‐binding residues or adjacent codons located in the exons 24 or 25 of the gene, a region considered a somatic hotspot [18]. In our study, molecular analysis identified a missense variant in the RNase IIIb domain in 13 tissue samples, confirming the pathogenic mechanism that results in an imbalance in the production of mature microRNAs in these tissues (Table 2).

In the two remaining samples, a PinB (P16) and a papillary thyroid microcarcinoma (PTMC) follicular variant (P8), no somatic variant was detected. Notably, distinct somatic variants were detected in different tissues from the same patient (P4, P5, and P10), demonstrating that the occurrence of the second hit is independent in each tissue and is not determined by the type of the germline variant [33].

These findings are consistent with the previously described mechanism of tumor development in *DICER1*‐related tumor predisposition, in which a LOF germline variant combined with a somatic variant in the wild‐type allele in the RNase IIIb domain still allows cell growth but under an altered miRNA profile, which promotes tumor development. Conversely, if the second acquired variant were also a LOF variant, the cell would be unable to survive and would proceed on the path to cell death [34]. In this context, the absence of a somatic hotspot variant in the PinB sample (P16) would support previous studies that demonstrate the mechanism of *DICER1* alteration in PinB is truly distinct from other *DICER1*‐related tumor predisposition‐associated tumors [35, 36]. In PinB, a germline truncating variant affects one allele and loss of heterozygosity (LOH) of the other allele is regularly found; in some cases, a second LOF alteration distributed throughout the gene has been described [37]. This reflects the exceptional situation in this tissue type; unlike in other tissues where the complete loss of the *DICER1* gene is incompatible with cell viability [34], the total loss of *DICER1* in the pineal gland still allows tumor development, behaving as a classic tumor suppressor in this tissue [14]. In this particular case (P16), it remains to be determined whether the mechanism of somatic alteration corresponds to LOH or to the uncommon case where the second somatic variant is a LOF variant dispersed throughout the gene.

In the case of P8, the absence of a somatic variant could be due to the small size of the lesion, making adequate sampling difficult and raising the possibility that the sample was not truly representative of tumor tissue. Therefore, the tumorigenesis mechanism in these two latter cases remains uncertain and additional studies are needed to clarify these findings. Additionally, an undetected epigenetic alteration, such as methylation‐mediated silencing of the remaining wild‐type allele, cannot be excluded as an alternative second‐hit mechanism in these tumors.

It should be noted that in all tumors a targeted molecular search for the corresponding germline variant was performed, and the variant was detected in every case.

Because the vast majority of tumors involved in this pathology primarily appear in children, the objective of offering molecular diagnosis to family members is to determine whether the variant is hereditary or not. This allows us to inform families about their potential risk of being carriers and developing disease‐related tumors. In this study, carrier status was ruled out in 30 relatives, ensuring that they do not have a hereditary risk of developing *DICER1*‐related tumors or transmitting the variant to their offspring. Conversely, individuals who are carriers of germline *DICER1* variants can enter follow‐up and surveillance protocols established at our hospital based on expert recommendations published to date [9, 38]. Accordingly, this study made it possible to identify several relatives who carried a pathogenic *DICER1* germline variant but had no clinical manifestations at the time of diagnosis.

It is worth mentioning that in P10, both the MNG and thyroid cancer were detected at early stages because her germline *DICER1* alteration was previously detected. Moreover, the carrier status of the index case′s brother was identified before he developed MNG; this enabled personalized medical follow‐up for this boy and the implementation of a surveillance protocol that allowed for early detection of MNG and performance of a preventive thyroidectomy, taking into account his sister′s history of TC. A similar approach was adopted for patient P17, who also underwent preventive thyroidectomy after early‐stage MNG was detected during surveillance for carriers of a pathogenic germline *DICER1* variant.

In P14, who was diagnosed with PTC, somatic analysis initially identified a hotspot variant and subsequently an associated germline variant. Although there was no clinically relevant history, molecular testing detected asymptomatic carriers within the family. Despite these favorable outcomes, caution is warranted, as phenotypic penetrance is highly variable even within the same family [6]. This means that many carriers will never develop tumorigenesis. Therefore, the risks and benefits of implementing these long‐term protocols must be carefully evaluated.

In this study we also included three samples of poorly differentiated TC (PDTC), a tumor type not frequently associated with *DICER1*‐related tumor predisposition. In all three tissue samples, molecular analysis detected a missense variant in the RNase IIIb domain. In one case, unfortunately peripheral blood was not available. In the two cases that the complete gene could be studied in peripheral blood (P18, P20), no pathogenic variant was detected, suggesting the somatic nature of the missense variants found in the tumors, although we cannot completely rule out constitutional mosaicism. These findings are consistent with previous evidence indicating that PDTC in infancy and childhood represents a specific manifestation of somatic DICER1‐related disease [19]. In turn, P20 had a diagnosis of neuroblastoma (NB), a tumor that by its presence alone does not suggest the presence of *DICER1*‐related tumor predisposition [18]. Although NB is not currently considered part of the established DICER1 tumor spectrum, isolated reports have raised the possibility of a rare or noncanonical association, warranting further molecular characterization before a causal relationship can be established [39, 40].

The limitations of our study include the following: 1) most samples referred to our laboratory for molecular diagnosis originated from the Endocrinology Service related to thyroid disorders; 2) for several families, complete clinical or family information could not be obtained and in some cases only the index case could be studied; 3) regarding tissue samples, molecular analysis could only be performed in a subset (14/25) of the tissues from index cases because of sample conservation issues and low yield or low quality of the extracted DNA; 4) the possible impact of the intronic variant corresponding to P7 on the splicing process was not analyzed since RNA sample was not available. Nevertheless, this variant has been previously reported in a patient with type III PPB [20].

## 5. Conclusion

We identified 12 novel germline variants of the *DICER1* gene (Figure 1), thereby expanding the existing body of molecular knowledge of this disorder and contributing to a deeper understanding of its causes and progression. Molecular testing enhances diagnostic precision and facilitates more effective follow‐up, enabling improved genetic counseling regarding tumor risk. The identification of carriers within families allowed the implementation of preventive measures for individuals who were asymptomatic at the time of diagnosis.

Importantly, the relevance of this study lies in providing valuable tools for the molecular analysis of genes implicated in early‐onset hereditary cancer in children. Such advances support earlier diagnostic and therapeutic interventions, ultimately improving life expectancy for both index cases and unaffected carriers. Nevertheless, the identification of asymptomatic carriers continues to raise complex ethical issues that remain unresolved and warrant further discussion.

## Funding

No funding was received for this manuscript.

## Ethics Statement

The study was approved by the Independent Ethics Committee of the “Prof. Dr. J. P. Garrahan” Pediatric Hospital (reference number: 830) on August 27, 2014. Written informed consent for the genetic study was obtained from all of the patients or their parents or guardians after a full explanation of the purpose and nature of all the procedures.

## Conflicts of Interest

The authors declare no conflicts of interest.

## Supporting Information

Additional supporting information can be found online in the Supporting Information section.

## Supporting information


**Supporting Information 1** Figure S1: Histological analysis of the lymph node in Patient 11 showed: A) Epitheliod granulomas (arrows) with scattered giant cells, lymphocytes, and stromal sclerosis (H&E 4X).

**Supporting Information 2** Figure S2: B) Numerous confluent nonnecrotizing granulomas (arrows) composed of epithelioid cells (H&E 20X).

## Data Availability

The data that support the findings of this study are available from the corresponding author upon reasonable request.
