# Supplementary material for: Expanding the Molecular Data of DICER1‐Related Tumor Predisposition: Novel Germline Variants in an Argentine Pediatric Cohort
Source: Hum Mutat. 2026 Sep 30;2026:3621830. doi: 10.1155/humu/3621830 (PMC13627890; doi:10.1155/humu/3621830)
Supplement: Supplementary file 1 [file HUMU-2026-3621830-s001.pdf]

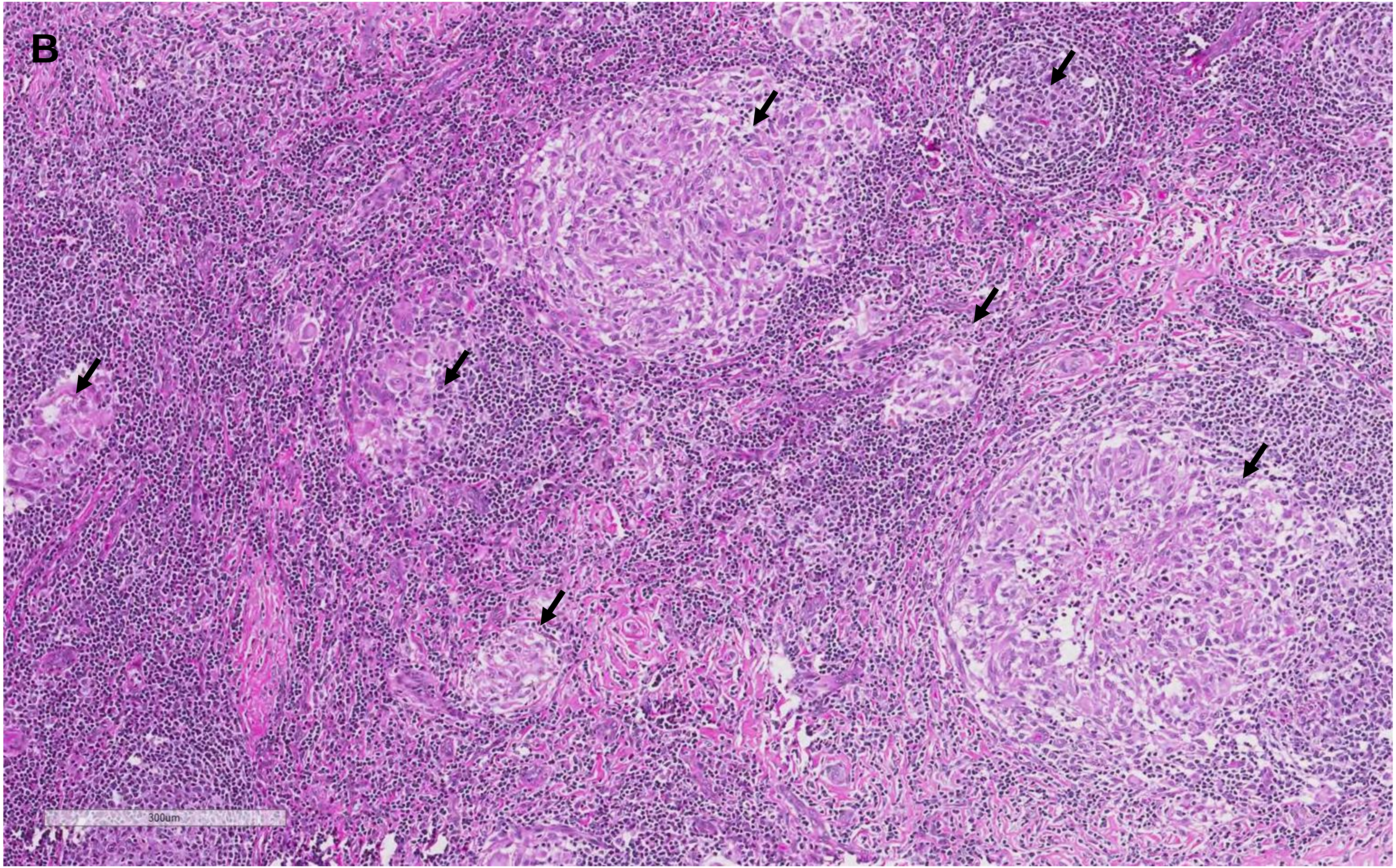
B
300um
